# MiRNA-181a Regulates Adipogenesis by Targeting Tumor Necrosis Factor-α (TNF-α) in the Porcine Model

**DOI:** 10.1371/journal.pone.0071568

**Published:** 2013-10-01

**Authors:** Hongyi Li, Xiao Chen, Lizeng Guan, Qien Qi, Gang Shu, Qingyan Jiang, Li Yuan, Qianyun Xi, Yongliang Zhang

**Affiliations:** 1 Guangdong Provincial Key Laboratory of Agro-animal Genomics and Molecular Breeding, ALLTECH-SCAU Animal Nutrition Control Research Alliance, South China Agricultural University, Guangzhou, China; 2 Fujian Provincial Key Laboratory of Preventive Veterinary Medicine and Biotechnology, College of Life Science, Longyan University, Fujian, China; 3 College of Life Science, Xiamen University, Xiamen, China; Georgia Regents University, United States of America

## Abstract

Adipogenesis is tightly regulated by altering gene expression, and TNF-α is a multifunctional cytokine that plays an important role in regulating lipogenesis. MicroRNAs are strong post-transcriptional regulators of cell differentiation. In our previous work, we found high expression of *miR-181a* in a fat-rich pig breed. Using bioinformatic analysis, *miR-181a* was identified as a potential regulator of TNF-α. Here, we validated TNF-α as the target of *miR-181a* by a dual luciferase assay. In response to adipogenesis, a mimic or inhibitor was used to overexpress or reduce *miR-181a* expression in porcine pre-adipocytes, which were then induced into mature adipocytes. Overexpression of *miR-181a* accelerated accumulation of lipid droplets, increased the amount of triglycerides, and repressed TNF-α protein expression, while the inhibitor had the opposite effect. At the same time, TNF-alpha rescued the increased lipogenesis by miR181a mimics. Additionally, *miR-181a* suppression decreased the expression of fatty synthesis associated genes *PDE3B* (phosphodiesterase 3B), LPL (lipoprotein lipase), *PPARγ* (proliferator-activated receptor-γ), *GLUT1*(glucose transporter), *GLUT4*, *adiponectin* and *FASN* (fatty acid synthase), as well as key lipolytic genes HSL (hormone-sensitive lipase) and *ATGL* (adipose triglyceride lipase) as revealed by quantitative real-time PCR. Our study provides the first evidence of the role of *miR-181a* in adipocyte differentiation by regulation of TNF-α, which may became a new therapeutic target for anti-obesity drugs.

## Introduction

Adipogenesis is a key process in adipocyte development and fat metabolism. Dysfunctions in adipocyte tissues may cause health problems such as obesity and coronary artery disease, both in humans [1] and companion animals [2]. On the other hand, adipose tissues are highly related to important aspects such as meat quality and animal productivity in farm animals [3]. Therefore, understanding the mechanisms regulating adipose tissue formation would be highly beneficial to both human and animal health.

Development from progenitor mesenchymal cells into adipocytes involves dramatic changes in gene expression programs. Adipogenesis in mammals is regulated both genetically and hormonally. It has been demosntrated that adipogenic transcription factors, such as proliferator-activated receptor-γ (PPARγ), CCAAT/enhancer-binding proteins (C/EBPs), Krüppel-like factors (KLFs) and sterol regulatory element-binding protein (SREBP) are involved in the differentiation of adipocytes [4–6].

Interestingly, microRNA (miR), a class of small non-coding RNA with multiple functions in regulating gene expression by targeting mRNA associated with the RNA-induced silencing complex (RISC) [7], are increasingly recognized for their involvement in adipogenesis regulation. Some miRs differentially expressed during adipogenesis have been identified, including *miR-24* [8,9], *miR-31* [9] and the *miR-17-92* cluster which alter cell proliferation [10]; *miR-8* which represses Wnt signaling [11]; and *miR-27* [12–14] and *miR-130* which target PPARγ [15]. Details on miRs regulating adipogenesis have been reviewed by Romao et al. [16].

TNF-α inhibits adipocyte differentiation from pre-adipocytes and mesenchymal stem cells [17,18] by downregulating the expression of key transcription factors for adipogenesis, such as C/EBPα and PPARγ, in pre-adipocytes [19,20]. It has been suggested that TNF-α triggers activation of NF-κB through the TAK1/TAB1/NIK axis, leading to a physical association between PPAR-γ and NF-κB, thereby inhibiting the ligand-dependent PPAR-γ transactivation [21]. It is also believed that TNF-α enhances the Wnt/b-catenin signaling pathway by inducing Msx2 expression, which in turn suppresses adipocytic differentiation [22]. MiRs, such as *miR-19a* and *miR-181* [23], have been shown to negatively regulate human TNF-α, but the regulation of TNF-α by miRs in adipocytes is still unclear. In our former study, *miR-181a* was shown to be significantly up-regulated in fat-rich pigs (Lantang, a local breed in China), relative to those with relatively less fat (Landrace), either in adipose tissue (Figure S1) or skeletal muscle. The results led to a hypothesis that *miR-181a* might play an important role in adipogenesis or adipocyte development. By using Targetscan and miRanda software, TNF-α was predicted to be a potential target for *miR-181a* in pigs and humans. In this study, we demonstrated the ability *miR-181a* to inhibit *TNF-α* expression by targeting the 3’ UTR of its mRNA, thus affecting adipogenesis.

## Materials and Methods

### Sample collection and culture of porcine primary pre-adipocytes

Subcutaneous fat tissue from a 7-day-old piglet was isolated aseptically and transferred to Dulbecco’s modified essential medium–F12 nutrient mixture, (DMEM/F12, GIBCO, New York, CA). After removing the visible connective tissues, the adipose tissue was cut into small pieces of about 1 mm^3^, and the subcutaneous pre-adipocytes were obtained as described in previous reports [24]. Minced tissue was transferred into a Carlsberg’s flask, digested in 0.2% type-II collagenase (1 mg/mL, GIBCO) for 2 h at 37°C and then filtered through a 150 µm mesh. Cells in the filtrate were centrifuged at 500 × *g* for 10 min, and erythrocytes were lysed using erythrocyte lysis buffer (0.154 MNH _4_Cl, 10 mM KHCO_3_ and 0.1 mM EDTA). After filtering through a 40 µm mesh, cells were rinsed with F12 and centrifuged at 1500 × *g* for 5 min. The pre-adipocytes were collected and plated in growth medium.

### Ethics Statement

All of the animal experiments were conducted in accordance with the guidelines of Guangdong Province on the Review of Welfare and Ethics of Laboratory Animals approved by the Guangdong Province Administration Office of Laboratory Animals (GPAOLA). All animal procedures were conducted under the protocol (SCAU-AEC-2010-0416) approved by the Animal Ethics Committee of South China Agricultural University.

### Target prediction and plasmid construction

Using Targetscan (www.targetscan.org) and miRanda (www.microrna.org), human *TNF-α* was predicted to be the target of *miR-181a*. By homology comparison, the pig *TNF-α* cDNA sequence was shown to have 82% identity with the human sequence. Importantly, the predicted target site is also conserved. The sequence of porcine *TNF-α* 3’ UTR of about 1140 bp was amplified from the pig genome using two primers, 5’- TCTAGACAGAGTGGGTATGCCAATGC-3’ and 5’- GTTAACACCAGTAGGGCGGTTACAGAC-3’. The PCR product was digested with *Xba*I and *Hpa*I and ligated to pGL3-control (Promega Co., Madison, WI) at the corresponding sites to obtain the plasmid pGL3-TNF-α-UTR. The mutant plasmid pGL3-TNF-α-UTR-muta was generated using the QuikChange Lightning site-directed mutagenesis kit (Stratagene, Santa Clara, USA) per the manufacturer’s instruction. In brief, the following primers for mutating the *miR-181a* binding site on the *TNF-α* UTR were designed (mutated nucleotides are underlined): 5’-TATTTATTTACTAGGACCGGTATTTATTCAGGAGGGCGAGG-3’ (forward) and 5’- CCTCGCCCTCCTGAATAAATACCGGTCCTAGTAAATAAATA-3’ (reverse). With pGL3-TNF-α-UTR as the template, the generated PCR product was then digested with *Dpn*I, transformed and amplified in *Escherichia coli* DH5α.

### Leuciferase reporter assay

CHO cells were maintained in RPMI 1640 (GIBCO) and supplemented with 10% fetal bovine serum (FBS, GIBCO). Cells were seeded in a 24-well plate one day before transfection with pGL3-TNF-α-UTR / pGL3-TNF-α-UTR-muta (500 ng), pRL-TK (Renilla luciferase normalization control, 50 ng, Promega) and *miR-181a* mimic (75 pM, GenePharma, Shanghai, China) using Lipofectamine 2000 ( and (Invitrogen Co., Carlsbad, CA), while a scrambled sequence as used as a negative control (NC). Cells were collected 48 h after transfection, and luciferase activity was measured using a dual luciferase reporter assay system (Promega).

### Cell culture and transfections

Porcine primary pre-adipocytes or Hela cells were maintained in DMEM-F12 and DMEM, respectively, with 10% FBS and cultured in a humidified 5% CO_2_ incubator with a constant temperature of 37 °C. Cells were seeded in 6-well plates the day before transfection and transfected with miR-181a mimic/inhibitor (100 pM) using Lipofectamine 2000, and the scrambled sequence was used as a negative control (NC group). The cells were harvested 48 h after transfection for Western blot analysis, and the pre-adipocytes were induced to differentiate 24 h after transfection as previously reported [24]. Cells were stimulated with insulin, dexamethasone, octoic acid and octoic acid (50 nM, Sigma, USA) for eight days and collected for quantitative realtime PCR (qRT-PCR), Oil Red O staining, triglyceride (TG) assay and Western blot.

### TNF-α siRNA design and transfections

SiTNF-α1(AGATTGAGGTGAAATCTTC), siTNF-α2(CTCAGATCATCGTCTCAAA), and siTNF-α3(GCCCAAGGACTCAGATCAT) were designed using siDesigner (http://sidirect2.rnai.jp/) [25] and synthesize by GenePharma(Shanghai, China). Pre-adipocytes were transfected with TNF-α siRNAs and the NC respectively, then cells was induced to differentiate as above described and collected at day eight for Oil Red O staining and TG assay.

### TNF-α inhibitor and TNF-α performance

Pre-adipocytes were added TNF-α inhibitor (Santa Cruz, California), TNF-α (Pepro Tech) or DMSO(NC), respectively, then induced to differentiate. Cells were cultured with such mixture medium for six days and collected for Oil Red O staining and TG assay.

### miR181a mimics and TNF-α performance

Pre-adipocytes were added miR181a mimics (100pM), TNF-α (Pepro Tech) and miR181a mimics + TNF-α, respectively, then induced to differentiate. Cells were cultured with such mixture medium for eight days and collected for Oil Red O staining and TG assay.

### RNA analysis and qRT-PCR

Total RNA was extracted from differentiated adipocytes using Trizol reagent (Invitrogen), and RNA density was determined with a radiometer (Eppendorf, Hamburg, Germany). Total RNA (1 µg) was reverse-transcribed into cDNA using the M-MLV reverse transcriptase (Promega) with OligodT18. After 1 h of incubation at 42°C and 10 min of deactivation at 75°C, the reaction mixes were used as the templates for PCR. qRT-PCR was performed with standard protocols on a STRATAGENE Mx3005P sequence detection system. The PCR mixture contained 1 µL of cDNA, 10 µL of 2× SYBR Green PCR Master Mix, 1.5 µM of each primer and water to make up the final volume to 20 µL. The reaction was performed in a 96-well optical plate at 95°C for 1 min, followed by 35 cycles of 95°C for 15 s, optimal reannealing temperature for 15 s, and 72°C for 40 s. All reactions were run in duplicate, and a negative control without template was included for each gene. Primers were designed based on the sequence of each gene by using Premier 5.0 (Table S1).

### MiR-181a qRT-PCR detection

Stem-loop qRT-PCR was performed as previously described [26]. A looped antisense primer (GTCGTATCCAGTGCGTGTCGTGGAGTCGGCAATTGCACTGGATACGACAACTCACC) was used for reverse transcription. The RT reaction mix was diluted to one tenth for use as the template for real-time PCR. The reaction was carried out in a 96-well optical plate at 95°C for 1 min, followed by 35 cycles of 95°C for 15 s, 56 °C for 15 s and 72°C for 25 s. The cycle threshold (Ct) was recorded, and the amount of miR-181a relative to that of U6 RNA was calculated using the expression 2^-(CtmiRNA2CtU6RNA)^.

### Oil Red O staining

Cells were rinsed with Ca^2+^, Mg^2+^-free phosphate-buffered saline (PBS) twice and fixed in 4% polyoxymethylene in PBS (w/v) for 30 min at room temperature. Oil Red O (0.5 g, Amresco Inc, solon, OH) was dissolved in isopropanol (100 ml, w/v), diluted with water (6:4, v/v) and filtered. The fixed cells were then stained with the filtered Oil Red O solution for 1 h at room temperature, washed in water and photographed.

### TG assay

Cells were washed with PBS and scraped from the plates in 100 µL lysis buffer per well. After being placed on ice for 5 min, the lysate was centrifuged at 8000 × *g*, 4°C for 1 min. The supernatant was analyzed in a TG assay using the Food Triglyceride Assay Kit (APLLYGEN, Beijin, China) according to the manufacturer’s protocol, with a series of diluted glycerol as a standard. Total protein detected by a bicinchoninic acid (BCA) assay (Bioteke, Beijin, China) was used for normalization of TG concentration.

### Western blot analysis

Cells were lysed by radio immunoprecipitation assay (RIPA) buffer with protease inhibitors. Total soluble protein was quantified using a BCA protein assay. Total protein (50 µg) was loaded onto a 10% SDS page gel, separated by electrophoresis and transferred onto a polyvinylidene difluoride (PVDF) membrane. Blots were blocked with 5% skim milk and incubated with the primary antibody overnight at 4°C, followed by incubation with the secondary antibody for 1 h at room temperature and measured with an Infrared Imaging System (LI-COR CO, Lincoln, NE). Protein expression was normalized by β-actin (Abcam, Cambridge, UK).

### Statistical analysis

Results are presented as means ± SEM, and all experiments included at least six replicates per group. Data were evaluated using Student’s *t*-test, and differences between groups were considered statistically significant at *P* < 0.05. All statistical analysis was performed with SPSS 17.0 software.

## Results

### Target verification of miR-181a against 3’ UTR of TNF-α mRNA using a luciferase report assay

**Figure 1:**
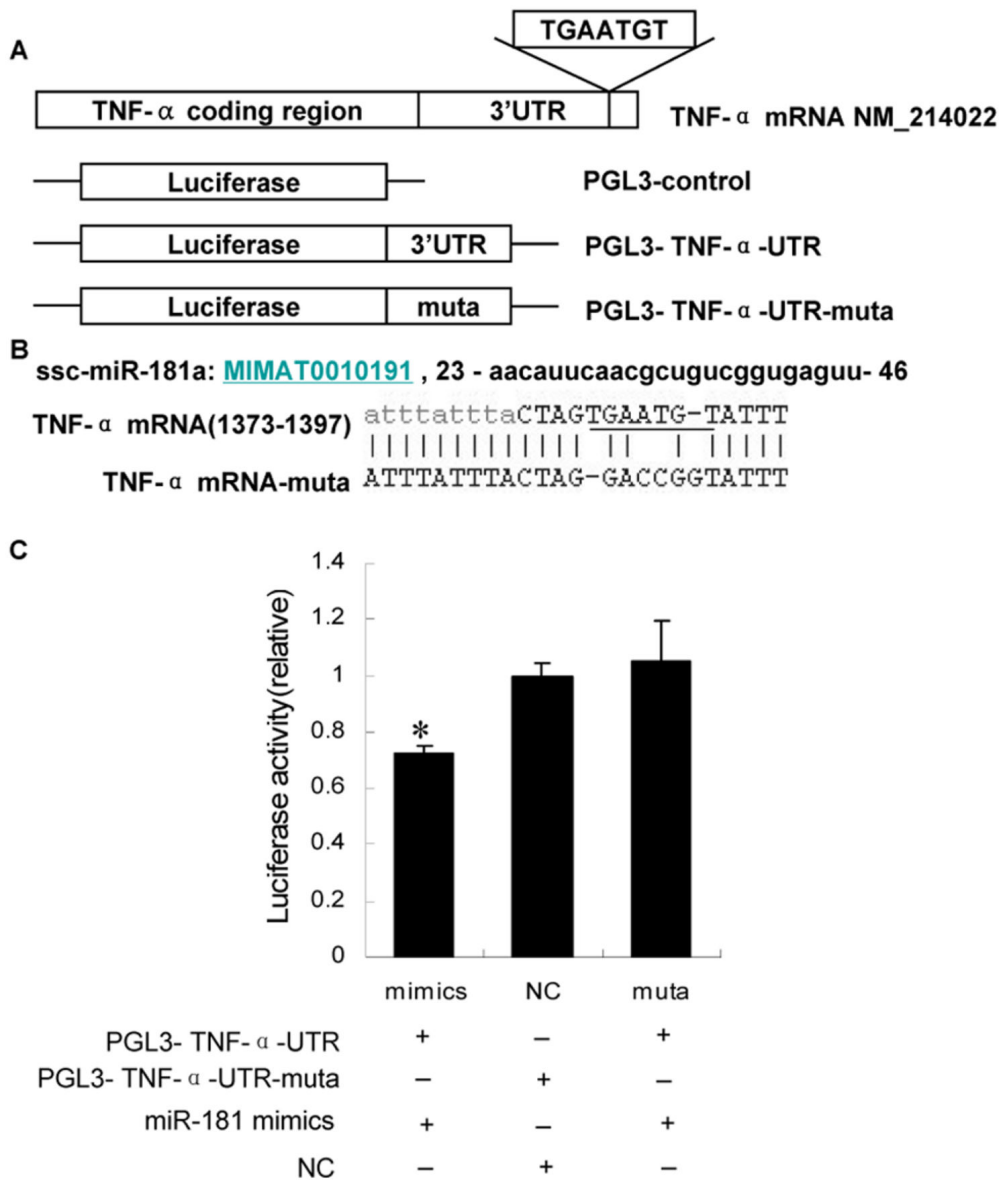
*MiR-181a* directly regulates TNF-α expression via 3’ UTR sites. (A) Schematic of *TNF-α* mRNA and the luciferase reporter plasmids containing the *miR-181a* binding sites of *TNF-α* mRNA. The 3’ UTR sites were inserted downstream of the luciferase reporter, as indicated. TGAATGT was the predicted target site of *miR-181a*. (B) *MiR-181a* sequences and predicted binding site between *miR-181a* and *TNF-α* mRNA. Sequence of *miR-181a* (www.mirbase.org) is shown. *TNF-α* mRNA has one putative binding site for *miR-181a* on the 3’ UTR. Seven nucleotides of *TNF-α* 3’ UTR (underlined) were replaced with GACCGGT using site-directed mutagenesis in order to disrupt the binding with *miR-181a* seed regions. (C) CHO cells were transfected with each of the constructed plasmids, together with *miR-181a* and Renilla luciferase reporter plasmid (*P < 0.05, n = 8).

To investigate whether *miR-181a* (Figure 1A) has an effect on adipogenesis or adipocyte development, target genes were predicted, and *miR-181a* was found to directly target *TNF-α* through its 3’-UTR sequence. The full-length 3’ UTR of *TNF-α* mRNA was inserted downstream of the luciferase gene in the pGL3 reporter plasmid, and the seed sequence was also mutated to disrupt *miR-181a* binding (Figure 1B). The wild-type (pGL3-TNF-α-UTR) or mutated (pGL3- TNF-α-UTR-muta) plasmid was co-transfected with the *miR-181a* mimic into CHO cells, together with the Renilla control pRL-TK for normalization. Forty eight hours after transfection, the luciferase activity of the *miR-181a* group was significantly lower than that of the NC group (*P* < 0,05), and the reduction was rescued in the mutation group (Figure 1C). Thus, TNF-α was initially confirmed as the target of *miR-181a*.

### MiR-181a Suppresses TNF-α Protein Levels in Hela Cells

**Figure 2:**
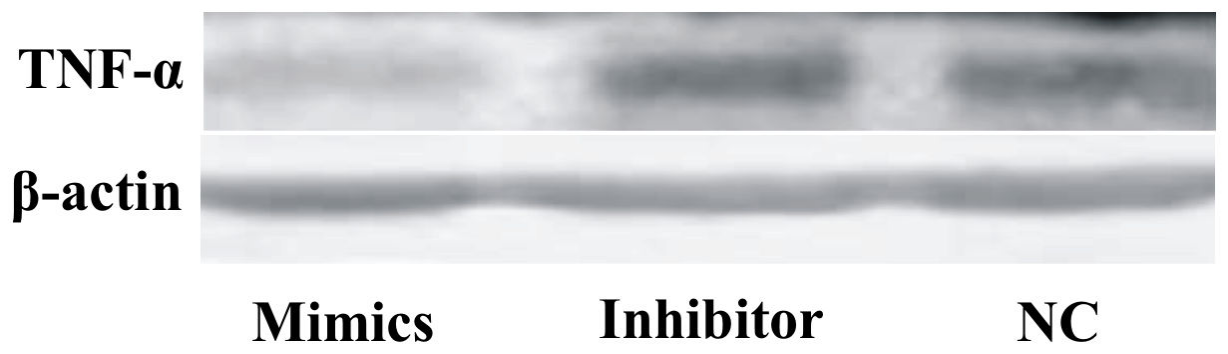
MiR-181aa suppresses TNF-α protein level in Hela cells detected by Western blot.

To further verify TNF-α as the target of *miR-181a*, we chose the TNF-α high expressing Hela cell line to detect whether overexpression of *miR-181a* could suppress the endogenous expression of TNF-α. *MiR-181a* mimic, inhibitor or NC was transfected into Hela cells and harvested 48 h after transfection. Western blot was performed to detect TNF-α protein levels. The *miR-181a* mimic suppressed the protein level of TNF-α as expected, while the inhibitor had the reverse effect (Figure 2), indicating that *miR-181a* indeedly targets TNF-α and regulates its endogenous expression in human cells.

### Porcine adipocyte differentiation model

**Figure 3:**
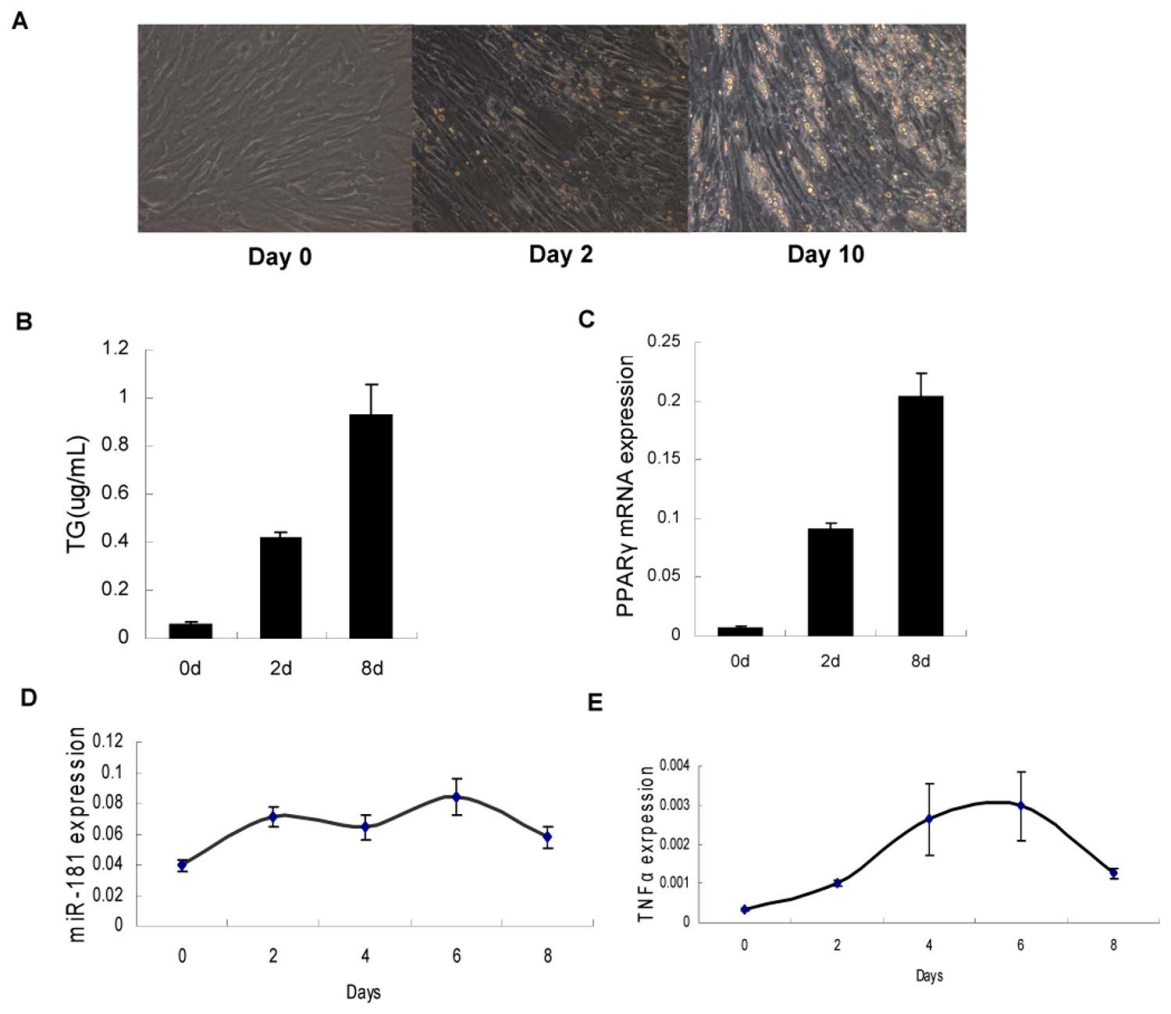
Porcine adipocyte differentiation model. During the process of differentiation, (A) lipid droplets gradually accumulated, (B) TG concentration increased dramatically on day 2 and day 8, measured as described in Materials and Methods. (B) *PPARγ* expression also increased during adipocyte differentiation, measured using qRT-PCR (n = 6). (D, E) Expression profile of *TNF* and *miR-181a* during differentiation of porcine adipocytes from day 0 to day 8, also measured using qRT-PCR (n = 6).

To determine the variations in *miR-181a* and *TNF-α* during the process of porcine adipogenesis, pre-adipocytes were obtained from a 7-day-old piglet and stimulated into mature adipocytes for about eight days. During this process, lipid droplets gradually accumulated (Figure 3A), while the TG concentration dramatically increased (Figure 3B). The expression of PPARγ, the classic marker of adipogenesis, also progressively increased (Figure 3C).

We measured the time-dependent decline in the expression levels of *miR-181a* (Figure 3D) and *TNF-α* (Figure 3E). *TNF-α* expression increased in the first few days, reached a peak value on day 6 and then decreased thereafter. The expression of *miR-181a* showed a trend similar to that of *TNF-α*, indicating that it may vary according to changes in *TNF-α*.

### Durability of miR-181a mimic in transfected porcine adipocytes

**Figure 4:**
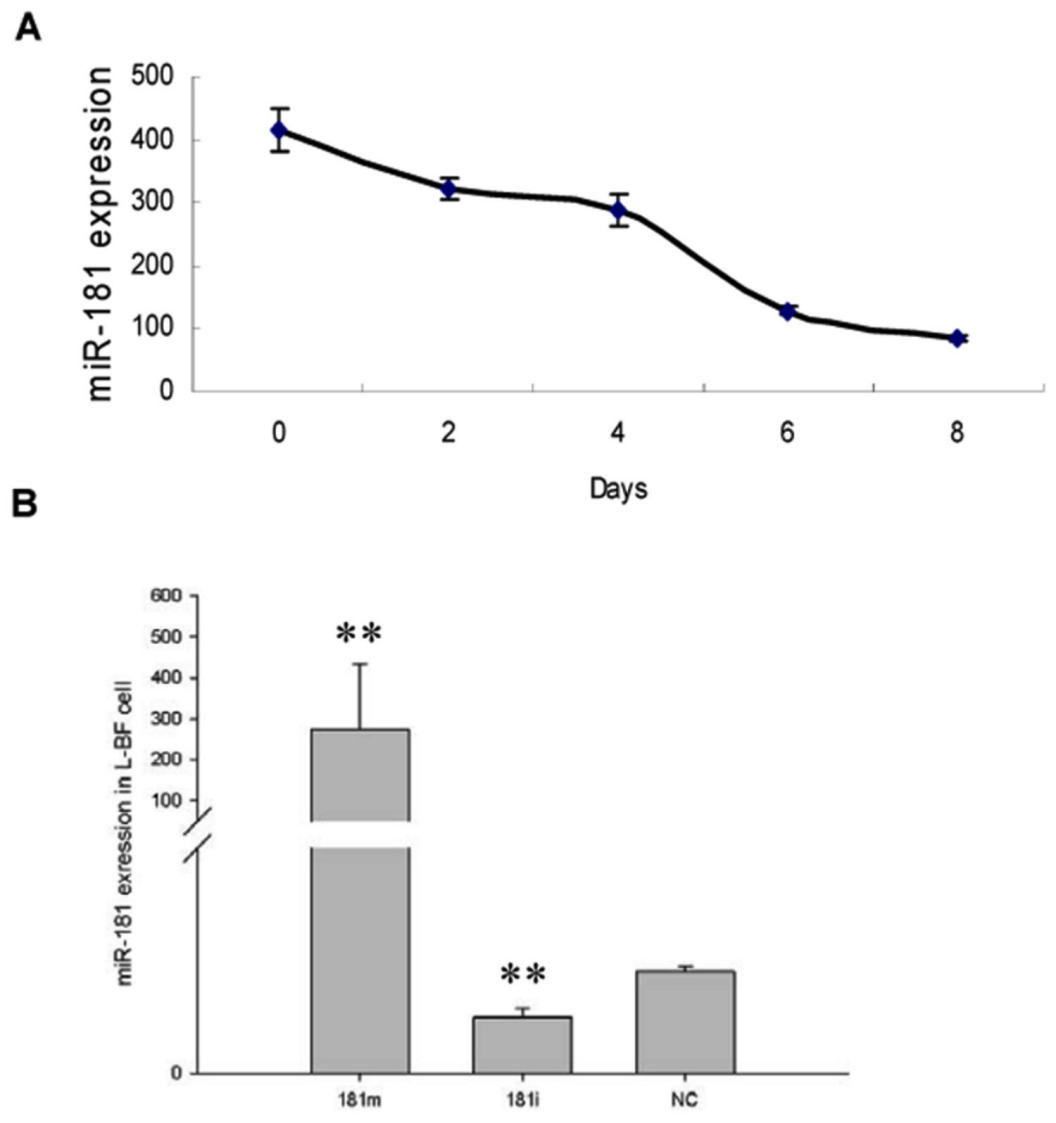
Level of *miR-181a* in porcine adipocytes after transfection of its mimic. The *miR-181a* mimic was transfected using Lipofectmine 2000, and endogenous *miR-181a* and mimics were quantified using qRT-PCR (n = 6). (A) Concentration of measured *miR-181a* in porcine adipocytes declined from day 0 to day 8. (B) At day 8, the measured *miR-181a* concentration in the *miR-181a* mimic transfected group remained significantly higher vs. control (*P* < 0.01), while transfection of the *miR-181a* inhibitor resulted in a significantly lower *miR-181a* level vs. the control (*P* < 0.01), n = 6.

In order to ensure effectiveness of the *miR-181a* mimic throughout the differentiation process, we verified its persistence after transfection. *MiR-181a* mimic, inhibitor or NC was transfected into pre-adipocytes, which were then induced to differentiate into mature adipocytes for 8 days (Figure 4A). Cells transfected with the mimic were harvested in 2-day intervals, while the inhibitor and NC groups were collected on day 8. Although the level of *miR-181a* gradually decreased during differentiation (Figure 4B), it was still dramatically higher than that of the NC group (*P* < 0.01), and the cells transfected with the inhibitor showed a lower *miR-181a* level than that of the NC group (*P* < 0.01, Figure 4C). These results suggest that use of the *miR-181a* mimic is feasible and suitable for further study.

### MiR-181a regulates adipocyte differentiation

**Figure 5:**
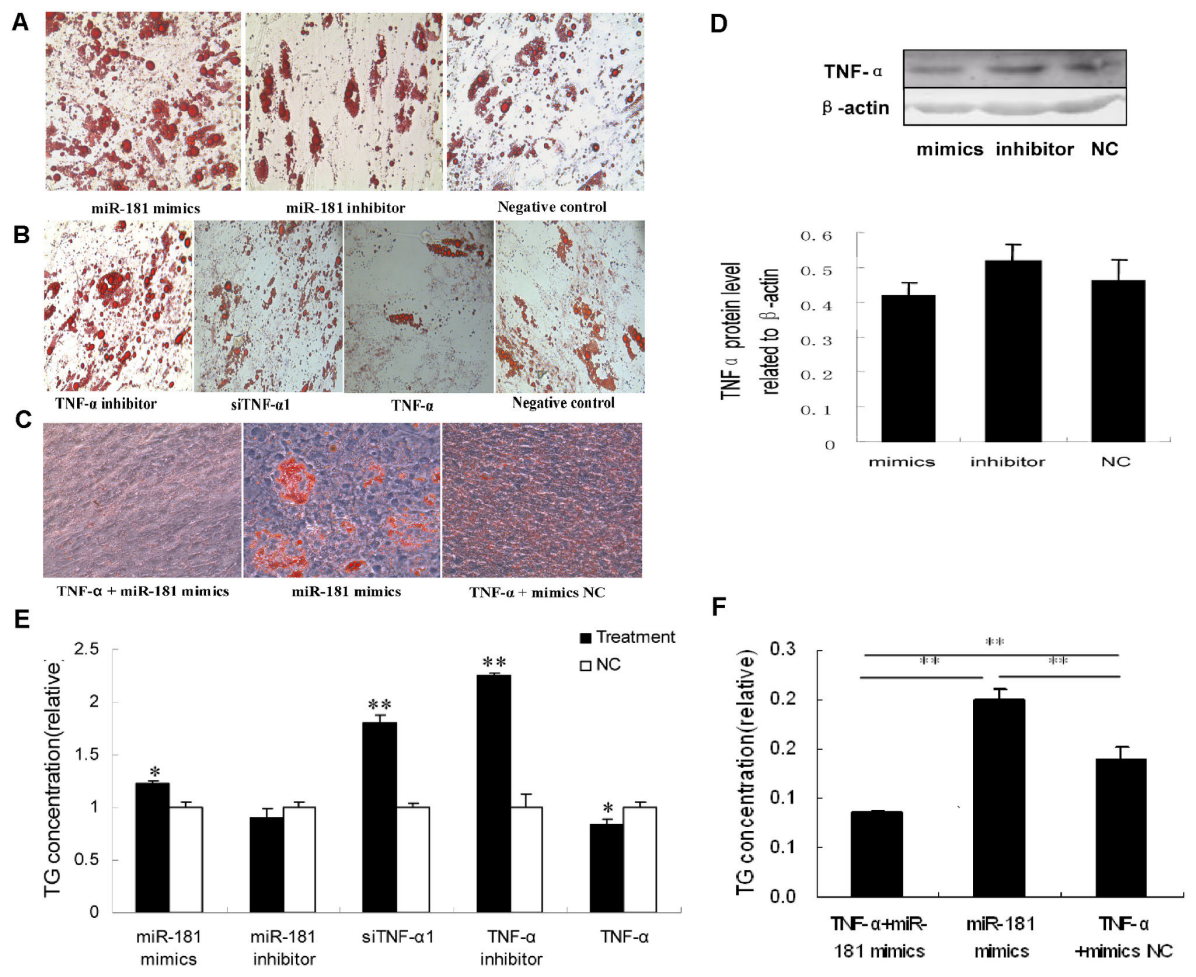
Changes in *miR-181a* levels modulate adipocyte differentiation. After transfection of protected *miR-181a* mimic or inhibitor, pre-adipocytes were stimulated to differentiate, taking TNF-α siRNA, TNF-α inhibitor and TNF-α as the reference or cotransfected controls. After 8 days, cells were harvested for analysis. (A) Formation of lipid droplets in the cells that transfected with miR-181a mimics and inhibitor were observed by staining with Oil Red O. (B) Formation of lipid droplets in the cells that treated with TNF-α siRNA, TNF-α inhibitor and TNF-α were observed as miR-181a treatment. (C) Formation of lipid droplets in the cells that contransfection with TNF-α were observed as miR-181a mimics treatment. (D) TNF-α protein abundance 8 days post-induction was assessed by Western blot analysis and quantified using gray scale scanning. (E,F) The degrees of differentiation in concorresponding to Figure 5B,5C treatment, respectively, were also determined by measuring the TG level, represented as the means SD, and each sample was assayed in triplicate (*P < 0.05, **P < 0.01, n=8).

To ascertain whether *miR-181a* has a direct effect on porcine adipocyte differentiation, the *miR-181a* mimic, inhibitor or NC was transfected into pre-adipocytes, taking TNF-α siRNA /inhibitor and TNF-α as the reference effects, and then stimulated to differentiate. After 8 days, formation of lipid droplets was observed by staining with Oil Red O. The *miR-181a* mimic obviously increased lipid droplets in porcine adipocytes (Figure 5A, 5C), same with the effects of TNF-α siRNA and TNF-α inhibitor (Figure 5B, 5C) and this regulation was rescued by the *miR-181a* inhibitor (Figure 5A) and TNF-α treatment (Figure 5B). But the inhibitor did not led to significant change of lipogenesis (Figure 5A) and TG content (Figure 5E) relative to control group. More interestingly, increased lipogenesis and TG content by miR-181a mimic dramatically diminished by TNF-α addition (Figure 5C, 5F). The degree of differentiation was also determined by measuring TG concentrations. Similar to the results of Oil Red O staining, the *miR-181a* mimic, TNF-α siRNA and TNF-α inhibitor could significantly increase the amount of TG (Figure 5E, 5F; *P < 0.05, **P < 0.01), and this effect was also rescued by transfection of the inhibitor and also TNF-α treatment (Figure 5E). By Western blot analysis on day 8, the *miR-181a* mimic decreased while the *miR-181a* inhibitor promoted TNF-α protein levels (Figure 5D).

### Transfection of miR-181a alters expression of fat metabolism related genes

**Figure 6:**
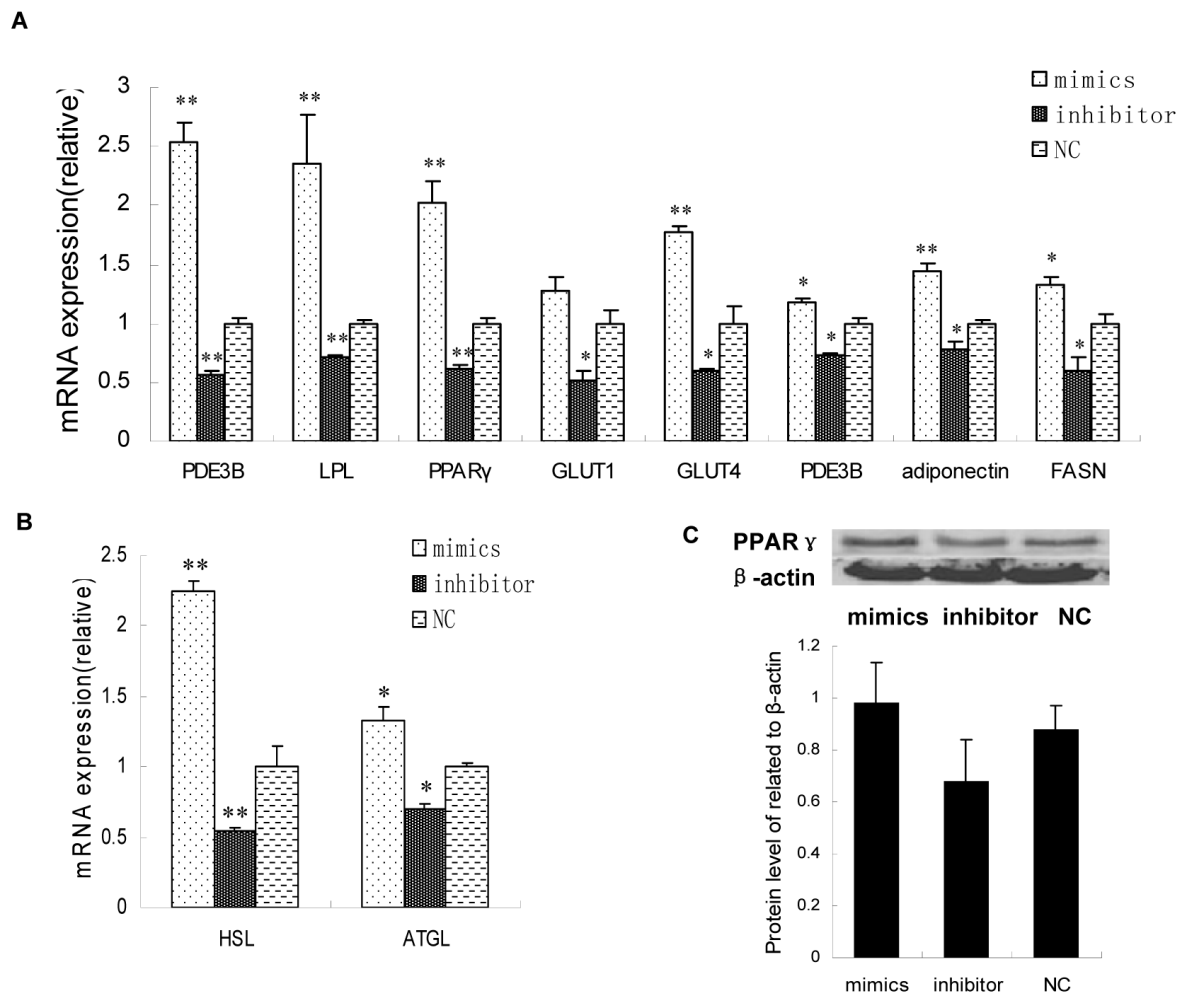
Fat metabolism related gene expression after transfection with *miR-181a* mimic and inhibitor. (A) Expression levels of *PDE3B*, *LPL*, *PPARγ*, *GLUT1*, *GLUT4*, *adiponectin* and *FASN* genes measured by qRT-PCR dramatically increased in cells after transfection of the *miR-181a* mimic vs. control, while they obviously decreased after transfection of inhibitor vs. control. Interestingly, (B) mRNA levels of *HSL* and *ATGL* increased after transfection with *miR-181a* and decreased after transfection with the inhibitor (*P < 0.05, **P < 0.01, n = 6). (C) Western blotting showed that the *miR-181a* may have increased protein levels of PPARγ.

To detect alterations in expression of fat metabolism related genes after *miR-181a* transfection, pre-adipocytes were prepared as described above and stimulated to differentiate for 8 days. Cells were collected to detect expression of the genes and proteins using qRT-PCR and Western blot, respectively. Expression levels of the fatty synthesis related genes (PDE3B, LPL, PPARγ, GLUT1, GLUT4, adiponectin and FASN) were dramatically increased in cells after transfection of the *miR-181a* mimic relative to control, while they decreased significantly after transfection of the inhibitor relative to the control (Figure 6A, *P < 0.05, **P < 0.01). Interestingly, mRNA levels of the lipolysis associated genes, *HSL* and *ATGL*, also increased after *miR-181a* transfection, and decreased after inhibitor transfection (Figure 6B, *P < 0.05, **P < 0.01). Western blotting showed that the *miR-181a* increased the protein levels of PPARγ (Figure 6C). We thus demonstrated that *miR-181a* promoted adipogenesis by targeting TNF-α and consequently altering the expression of genes regulated by TNF-α.

## Discussion

Adipocyte development and fat metabolism are important topics of research as dysfunctions of adipocyte tissue are associated with health problems. Some miRNAs have been reported to suppress adipocyte differentiation. For example, the *miR-27* family negatively regulates adipogenesis by repressing PPARγ protein levels [12,14]. By binding to the same target, *miR-130* is also a repressor as it inhibits induction of pre-adipocytes into mature adipocytes [15]. Likewise, *miR-448* and *miR-15a* were reported as potential inhibitors of adipogenesis, by suppressing Kruppel-like factor 5 (KLF5) [27] and promoting proliferation [28], respectively, while other miRNAs can accelerate adipocyte differentiate, such as *miR-103* and *miR-143* [29]. Thus, a greater understanding of miRNAs involved in adipogenesis will be beneficial for the exploration of new mechanisms in adiopcyte development and its regulation.

Apart from key regulators such as PPARγ, *in vivo* studies have suggested that TNF-α is relevant to adipocyte metabolism, including its effects on glucose homeostasis [30], promotion of lipolysis [31] and potent inhibition of adipocyte differentiation [32,33]. The variation in *TNF-α* expression, peaking on day 6, during primary porcine adipocyte development suggests a role in lipogenesis.

This study demonstrates that *miR-181a* is a novel regulator of *TNF-α* in porcine adipocytes. Most miRNAs function by targeting the 3’ UTR of mRNA, although a few miRNAs have been demonstrated to regulate gene expression through the coding region of target mRNAs [8,34]. Results of our bioinformatic analysis and luciferase reporter assay showed that *miR-181a* also functions by targeting the 3’ UTR of *TNF-α* mRNA with its seed region (Figure 1C). In both Hela cells and primary porcine adipocytes tested in our study, the *miR-181a* mimic decreased the TNF-α protein level, which was rescued by the *miR-181a* inhibitor. These results provide strong evidence that *miR-181a* inhibits TNF-α both in human and porcine cells. Since TNF-α is believed to inhibit adipocyte differentiation (27), it follows that suppression of TNF-α should promote this process. Here we showed forced expression of *miR-181a* via transfection of its mimic in cultured pocine primary adipocytes resulted in increased lipid droplets and TG levels. Moreover, pre-adipocytes transfected with TNF-α siRNA or deal with TNF-α inhibitor showed the similar result of *miR-181a* transfection, increasing lipid droplets and TG levels (Figure 5B, 5E, 5C, 5F). Moreover, TNF-α neutralized the effect of lipogenesis caused by miR181a mimics (Figure 5C, 5F). The combined results above strongly suggested that miR181a possibally regualtes porcine lipogenesis by targeting TNF-α.

Though the miR181a inhibitor rescued the increased lipogenesis by addition of miR181a mimics, the inhibitor did not cause significant change of lipogenesis relative to control group. Similarly, the miR181a inhibitor rescued the increased lipogenesis by addition of siRNA-TNF-alpha, but the inhibitor had no statistical effect on the lipogenesis (Data not shown), There may be a risk of off-target effects because the inhibitor can sequester the miRNA without causing degradation [35], which was described by previous studies [36,37]. Therefore, measuring miRNA levels is sometimes not a reliable measure of miRNA inhibition [38].

Although several targets of *miR-181a* have been verified, such as the homeobox protein Hox-A11, a repressor of muscle differentiation [39], p300/CBP-associated factor (PCAF) [40] and GRP78 [41], targets related to adipocyte differentiation have not yet been reported. For the first time we demonstrated that *miR-181a* targeted the *TNF-α* genes in association with porcine adipogensis. During the preparation of this manuscript, Río et al. also verified *TNF-α* as the target of human *miR-181c* in hematopoietic progenitor cells [42].

Data on the effect of miRNAs on TNF-α during adipgenesis is limited. It has been reported that TNF-α downregulates the expression of *miR-103* and *miR-143* in differentiated adipocytes [29], but the direct relationships between these two miRNA and TNF-α remain unclear. Liu et al. indicated that human TNF-α is a novel target of *miR-19a* [23], which is consistent with our result (S2). The present study is the first to indicate that *miR-181a* promotes adipogenesis via targeting TNF-α.

TNF-α has been demonstrated to regulate adipocyte genes such as by downregulating expression of genes involved in the uptake and storage of FFA and glucose associated genes *PPARγ*, *GLUT4*, *LPL* and *FASN* [43], the lipolytic genes *HSL* [44] and *ATGL* [45], and the TNF-α signaling gene *PDE3B*. By qRT-PCR analysis, this study showed that *miR-181a* increased the expression of those genes listed above (Figure 6A), potentially by reducing TNF-α levels.

PPARγ was the first transcriptional factor found to target TNF-α signaling in adipocytes and plays a key role in promoting adipogenesis. It was proposed that TNF-α inhibits PPARγ by activating NFκB through the NIK-TAK1/TAB1-mediated cascade [21]. Since *PPARγ* mRNA dramatically increased and its protein level also increased after *miR-181a* transfection, it is conceivable that the observed changes in expression of genes such as *GLUT4* may be, at least partly, due to alteration of *PPARγ* expression. As the GLUT4 promoter includes response elements for PPARγ and is regulated by PPARγ [46], it is likely that *miR-181* increased the expression of GLUT4 via a TNF-α targeting and PPARγ dependent mechanism. LPL is a triglyceride hydrolase which hydrolyses plasma lipoproteins to provide FFA for adipocyte storage and lipogenesis. It was reported that TNF-α suppresses LPL in 3T3-L1 adipocytes [44]. In this study, *miR-181a* was shown to increase *LPL* mRNA expression by inhibiting TNF-α. Nevertheless, LPL is also regulated by PPARγ, and therefore the influence of *miR-181* on LPL expression by repressing TNF-α may be mediated primarily through elevation of PPARγ expression [47].

Although HSL is a regulator of stimulus-induced lipolysis [47], a previous study showed that TNF-α represses *HSL* mRNA expression [43]. Thus it is reasonable that *miR-181a* elevated *HSL* mRNA expression by inhibiting TNF-α in our study. ATGL, a rate-limiting enzyme that mediates basal lipolysis, is also negatively regulated by TNF-α (44), and its mRNA level was shown in our study to be upregulated by *miR-181a*. In addition, our results showed that inhibition of *miR-181a* decreased *PDE3B* mRNA expression by increasing TNF-α expression, which is in line with a former report that *TNF-α* promotes perilipin phosphorylation by decreasing the expression of PDE3B, resulting in accelerating TG hydrolysis [48].

Obesity is a worldwide health problem and is a major risk factor for chronic diseases. It is characterized by the increased number and expanded size of adipocytes [49]. It is known that obesity is associated with elevated infiltration of macrophages into adipose tissue [50,51], which are principally responsible for the increased production of adipose tissue-derived TNF-α [52]. The pig is a useful model organism for comparative studies [53]. Since we demonstrated *miR-181a* as a new regulator of TNF-α in porcine adipocytes, suppression of *miR-181a* expresssion may lead to inhibition of adipocyte differentiation. Therefore, *miR-181a* may be a new biomarker or new potent therapeutic target for obesity.

In summary, *miR-181a* regulates adipogenesis by affecting expression of TNF-α, as well as genes involved in adipogenesis. This paper contributes to our understanding of adipogenesis regulation via a miRNA and TNF-α mediated pathway.

## Supporting Information

Figure S1 **Expression of *miR-181a* in different breed of pigs.** Total RNA were extracted from adipose tissues of Landrace and Lantang pigs (Fat-rich pigs) and subjected to miRNA sequencing and microarray.
(PDF)

Table S1 **The primers of fat metabolism related genes.**
(DOC)

## References

[1] Poulos SP, Hausman DB, Hausman GJ (2010) The development and endocrine functions of adipose tissue. Mol Cell Endocrinol 323: 20-34. doi:10.1016/j.mce.2009.12.011. PubMed: 20025936. 20025936 10.1016/j.mce.2009.12.011

[2] German AJ, Ryan VH, German AC, Wood IS, Trayhurn P (2010) Obesity, its associated disorders and the role of inflammatory adipokines in companion animals. Vet J 185: 4-9. doi:10.1016/j.tvjl.2010.04.004. PubMed: 20472476. 20472476 10.1016/j.tvjl.2010.04.004

[3] Dodson MV, Jiang Z, Chen J, Hausman GJ, Guan le L et al. (2010) Allied industry approaches to alter intramuscular fat content and composition in beef animals. J Food Sci 75: R1-R8. doi:10.1111/j.1750-3841.2009.01382.x. PubMed: 20492190. 20492190 10.1111/j.1750-3841.2009.01396.x

[4] Shao D, Lazar MA (1997) Peroxisome proliferator activated receptor gamma, CCAAT/enhancer-binding protein alpha, and cell cycle status regulate the commitment to adipocyte differentiation. J Biol Chem 272: 21473-21478. doi:10.1074/jbc.272.34.21473. PubMed: 9261165. 9261165 10.1074/jbc.272.34.21473

[5] Rosen ED, Walkey CJ, Puigserver P, Spiegelman BM (2000) Transcriptional regulation of adipogenesis. Genes Dev 14: 1293-1307. PubMed: 10837022. 10837022

[6] White UA, JM S (2010) Transcriptional factors that promote formation of white adipose tissue. Mol Endocrinol: Cell and Publishing House 318: 10-14

[7] Bartel DP (2009) MicroRNAs: target recognition and regulatory functions. Cell 136: 215-233. doi:10.1016/j.cell.2009.01.002. PubMed: 19167326. 19167326 10.1016/j.cell.2009.01.002 PMC3794896

[8] Lal A, Kim HH, Abdelmohsen K, Kuwano Y, Pullmann R Jr. et al. (2008) p16(INK4a) translation suppressed by miR-24. PLOS ONE 3: e1864. doi:10.1371/journal.pone.0001864. PubMed: 18365017. 18365017 10.1371/journal.pone.0001864 PMC2274865

[9] Sun F, Wang J, Pan Q, Yu Y, Zhang Y et al. (2009) Characterization of function and regulation of miR-24-1 and miR-31. Biochem Biophys Res Commun 380: 660-665. doi:10.1016/j.bbrc.2009.01.161. PubMed: 19285018. 19285018 10.1016/j.bbrc.2009.01.161

[10] Wang Q, Li YC, Wang J, Kong J, Qi Y et al. (2008) miR-17-92 cluster accelerates adipocyte differentiation by negatively regulating tumor-suppressor Rb2/p130. Proc Natl Acad Sci U S A 105: 2889-2894. doi:10.1073/pnas.0800178105. PubMed: 18287052. 18287052 10.1073/pnas.0800178105 PMC2268555

[11] Kennell JA, Gerin I, MacDougald OA, Cadigan KM (2008) The microRNA miR-8 is a conserved negative regulator of Wnt signaling. Proc Natl Acad Sci USA 105: 15417-15422. doi:10.1073/pnas.0807763105. PubMed: 18824696. 18824696 10.1073/pnas.0807763105 PMC2563117

[12] Karbiener M, Fischer C, Nowitsch S, Opriessnig P, Papak C et al. (2009) microRNA miR-27b impairs human adipocyte differentiation and targets PPARgamma. Biochem Biophys Res Commun 390: 247-251. doi:10.1016/j.bbrc.2009.09.098. PubMed: 19800867. 19800867 10.1016/j.bbrc.2009.09.098

[13] Kim SY, Kim AY, Lee HW, Son YH, Lee GY et al. (2010) miR-27a is a negative regulator of adipocyte differentiation via suppressing PPARgamma expression. Biochem Biophys Res Commun 392: 323-328. doi:10.1016/j.bbrc.2010.01.012. PubMed: 20060380. 20060380 10.1016/j.bbrc.2010.01.012

[14] Lin Q, Gao Z, Alarcon RM, Ye J, Yun Z (2009) A role of miR-27 in the regulation of adipogenesis. FEBS J 276: 2348-2358. doi:10.1111/j.1742-4658.2009.06967.x. PubMed: 19348006. 19348006 10.1111/j.1742-4658.2009.06967.x PMC5330386

[15] Lee EK, Lee MJ, Abdelmohsen K, Kim W, Kim MM et al. (2010) miR-130 suppresses adipogenesis by inhibiting peroxisome proliferator-activated receptor gamma expression. Mol Cell Biol 31: 626-638. PubMed: 21135128. 21135128 10.1128/MCB.00894-10 PMC3028659

[16] Romao JM, Jin W, Dodson MV, Hausman GJ, Moore SS et al. (2011) MicroRNA regulation in mammalian adipogenesis. Exp Biol Med (Maywood) 236: 997-1004. doi:10.1258/ebm.2011.011101. PubMed: 21844119. 21844119 10.1258/ebm.2011.011101

[17] Xu H, Sethi JK, Hotamisligil GS (1999) Transmembrane tumor necrosis factor (TNF)-alpha inhibits adipocyte differentiation by selectively activating TNF receptor 1. J Biol Chem 274: 26287-26295. doi:10.1074/jbc.274.37.26287. PubMed: 10473584. 10473584 10.1074/jbc.274.37.26287

[18] Takada I, Kouzmenko AP, Kato S (2009) Molecular switching of osteoblastogenesis versus adipogenesis: implications for targeted therapies. Expert Opin Ther Targets 13: 593-603. doi:10.1517/14728220902915310. PubMed: 19397478. 19397478 10.1517/14728220902915310

[19] Xing H, Northrop JP, Grove JR, Kilpatrick KE, Su JL et al. (1997) TNF alpha-mediated inhibition and reversal of adipocyte differentiation is accompanied by suppressed expression of PPARgamma without effects on Pref-1 expression. Endocrinology 138: 2776-2783. doi:10.1210/en.138.7.2776. PubMed: 9202217. 9202217 10.1210/endo.138.7.5242

[20] Loftus TM, Lane MD (1997) Modulating the transcriptional control of adipogenesis. Curr Opin Genet Dev 7: 603-608. doi:10.1016/S0959-437X(97)80006-8. PubMed: 9388775. 9388775 10.1016/s0959-437x(97)80006-8

[21] Suzawa M, Takada I, Yanagisawa J, Ohtake F, Ogawa S et al. (2003) Cytokines suppress adipogenesis and PPAR-gamma function through the TAK1/TAB1/NIK cascade. Nat Cell Biol 5: 224-230. doi:10.1038/ncb942. PubMed: 12598905. 12598905 10.1038/ncb942

[22] Qadir AS, Lee HL, Baek KH, Park HJ, Woo KM et al. (2011) Msx2 is required for TNF-alpha-induced canonical Wnt signaling in 3T3-L1 preadipocytes. Biochem Biophys Res Commun 408: 399-404. doi:10.1016/j.bbrc.2011.04.029. PubMed: 21514273. 21514273 10.1016/j.bbrc.2011.04.029

[23] Liu M, Wang Z, Yang S, Zhang W, He S et al. (2011) TNF-α is a novel target of miR-19a. Int J Oncol 38: 1013-1022. PubMed: 21271217. 21271217 10.3892/ijo.2011.924

[24] Zhou G, Wang S, Wang Z, Zhu X, Shu G et al. (2010) Global comparison of gene expression profiles between intramuscular and subcutaneous adipocytes of neonatal landrace pig using microarray. Meat Sci 86: 440-450. doi:10.1016/j.meatsci.2010.05.031. PubMed: 20573458. 20573458 10.1016/j.meatsci.2010.05.031

[25] Naito Y, Yamada T, Ui-Tei K, Morishita S, Saigo K (2004) siDirect: highly effective, target-specific siRNA design software for mammalian RNA interference. Nucleic Acids Res 32: W124-W129. doi:10.1093/nar/gkh442. PubMed: 15215364. 15215364 10.1093/nar/gkh442 PMC441580

[26] Chen C, Ridzon DA, Broomer AJ, Zhou Z, Lee DH et al. (2005) Real-time quantification of microRNAs by stem-loop RT-PCR. Nucleic Acids Res 33: e179. doi:10.1093/nar/gni178. PubMed: 16314309. 16314309 10.1093/nar/gni178 PMC1292995

[27] Kinoshita M, Ono K, Horie T, Nagao K, Nishi H et al. (2010) Regulation of adipocyte differentiation by activation of serotonin (5-HT) receptors 5-HT2AR and 5-HT2CR and involvement of microRNA-448-mediated repression of KLF5. Mol Endocrinol 24: 1978-1987. doi:10.1210/me.2010-0054. PubMed: 20719859. 20719859 10.1210/me.2010-0054 PMC5417392

[28] Andersen DC, Jensen CH, Schneider M, Nossent AY, Eskildsen T et al. (2010) MicroRNA-15a fine-tunes the level of Delta-like 1 homolog (DLK1) in proliferating 3T3-L1 preadipocytes. Exp Cell Res 316: 1681-1691. doi:10.1016/j.yexcr.2010.04.002. PubMed: 20385127. 20385127 10.1016/j.yexcr.2010.04.002

[29] Xie H, Lim B, Lodish HF (2009) MicroRNAs induced during adipogenesis that accelerate fat cell development are downregulated in obesity. Diabetes 58: 1050-1057. doi:10.2337/db08-1299. PubMed: 19188425. 19188425 10.2337/db08-1299 PMC2671055

[30] Stephens JM, Pekala PH (1991) Transcriptional repression of the GLUT4 and C/EBP genes in 3T3-L1 adipocytes by tumor necrosis factor-alpha. J Biol Chem 266: 21839-21845. PubMed: 1939208. 1939208

[31] Kawakami M, Murase T, Ogawa H, Ishibashi S, Mori N et al. (1987) Human recombinant TNF suppresses lipoprotein lipase activity and stimulates lipolysis in 3T3-L1 cells. J Biochem 101: 331-338. PubMed: 3495531. 3495531 10.1093/oxfordjournals.jbchem.a121917

[32] Beutler B, Greenwald D, Hulmes JD, Chang M, Pan YC et al. (1985) Identity of tumour necrosis factor and the macrophage-secreted factor cachectin. Nature 316: 552-554. doi:10.1038/316552a0. PubMed: 2993897. 2993897 10.1038/316552a0

[33] Torti FM, Dieckmann B, Beutler B, Cerami A, Ringold GM (1985) A macrophage factor inhibits adipocyte gene expression: an in vitro model of cachexia. Science 229: 867-869. doi:10.1126/science.3839597. PubMed: 3839597. 3839597 10.1126/science.3839597

[34] Eulalio A, Huntzinger E, Izaurralde E (2008) Getting to the root of miRNA-mediated gene silencing. Cell 132: 9-14. doi:10.1016/j.cell.2007.12.024. PubMed: 18191211. 18191211 10.1016/j.cell.2007.12.024

[35] Esau C, Davis S, Murray SF, Yu XX, Pandey SK et al. (2006) miR-122 regulation of lipid metabolism revealed by in vivo antisense targeting. Cell Metab 3: 87-98. doi:10.1016/j.cmet.2006.01.005. PubMed: 16459310. 16459310 10.1016/j.cmet.2006.01.005

[36] Xie YF, Shu R, Jiang SY, Liu DL, Ni J et al. (2013) MicroRNA-146 inhibits pro-inflammatory cytokine secretion through IL-1 receptor-associated kinase 1 in human gingival fibroblasts. J Inflamm (Lond) 10: 20. doi:10.1186/1476-9255-10-20. PubMed: 23680172. 23680172 10.1186/1476-9255-10-20 PMC3660163

[37] Stenvang J, Petri A, Lindow M, Obad S, Kauppinen S (2012). nhib of MicroRNA Funct By antimiR Oligonucleotides Silence 3: 1. 10.1186/1758-907X-3-1 PMC3306207 22230293

[38] Wang Z (2009) MicroRNA interference technologies: 7 Anti-miRNA Antisense Oligonucleotides Technology 460 Springer Verlag pp. 127-143.

[39] Naguibneva I, Ameyar-Zazoua M, Polesskaya A, Ait-Si-Ali S, Groisman R, et al (2006) The microRNA miR-181 targets the homeobox protein Hox-A11 during mammalian myoblast differentiation. Nat Cell Biol 8: 278-284. doi:10.1038/ncb1373. PubMed: 16489342. 16489342 10.1038/ncb1373

[40] Zhao J, Gong AY, Zhou R, Liu J, Eischeid AN et al. (2012) Downregulation of PCAF by miR-181a/b provides feedback regulation to TNF-alpha-induced transcription of proinflammatory genes in liver epithelial cells. J Immunol 188: 1266-1274. doi:10.4049/jimmunol.1101976. PubMed: 22219331. 22219331 10.4049/jimmunol.1101976 PMC3262895

[41] Ouyang YB, Lu Y, Yue S, Xu LJ, Xiong XX et al. (2012) miR-181 regulates GRP78 and influences outcome from cerebral ischemia in vitro and in vivo. Neurobiol Dis 45: 555-563. doi:10.1016/j.nbd.2011.09.012. PubMed: 21983159. 21983159 10.1016/j.nbd.2011.09.012 PMC3251314

[42] Rio P, Agirre X, Garate L, Banos R, Alvarez L et al. (2012) Downregulated expression of hsa-miR-181c in Fanconi anemia patients: implications in TNFalpha regulation and proliferation of hematopoietic progenitor cells. Blood. 10.1182/blood-2011-01-331017 22310912

[43] Ruan H, Miles PD, Ladd CM, Ross K, Golub TR et al. (2002) Profiling gene transcription in vivo reveals adipose tissue as an immediate target of tumor necrosis factor-alpha: implications for insulin resistance. Diabetes 51: 3176-3188. doi:10.2337/diabetes.51.11.3176. PubMed: 12401708. 12401708 10.2337/diabetes.51.11.3176

[44] Ruan H (2003) Troglitazone Antagonizes Tumor Necrosis Factor- -induced Reprogramming of Adipocyte Gene Expression by Inhibiting the Transcriptional Regulatory Functions of NF-B. J Biol Chem 278: 28181-28192. doi:10.1074/jbc.M303141200. PubMed: 12732648. 12732648 10.1074/jbc.M303141200

[45] Kralisch S, Klein J, Lossner U, Bluher M, Paschke R et al. (2005) Isoproterenol, TNFalpha, and insulin downregulate adipose triglyceride lipase in 3T3-L1 adipocytes. Mol Cell Endocrinol 240: 43-49. doi:10.1016/j.mce.2005.06.002. PubMed: 16009485. 16009485 10.1016/j.mce.2005.06.002

[46] Jain RG, Phelps KD, Pekala PH (1999) Tumor necrosis factor-alpha initiated signal transduction in 3T3-L1 adipocytes. J Cell Physiol 179: 58-66. doi:10.1002/(SICI)1097-4652(199904)179:1. PubMed: 10082133. 10082133 10.1002/(SICI)1097-4652(199904)179:1<58::AID-JCP8>3.0.CO;2-1

[47] Cawthorn WP, Sethi JK (2008) TNF-α and adipocyte biology. FEBS Lett 582: 117-131. doi:10.1016/j.febslet.2007.11.051. PubMed: 18037376. 18037376 10.1016/j.febslet.2007.11.051 PMC4304634

[48] Zhang HH, Halbleib M, Ahmad F, Manganiello VC, Greenberg AS (2002) Tumor necrosis factor-alpha stimulates lipolysis in differentiated human adipocytes through activation of extracellular signal-related kinase and elevation of intracellular cAMP. Diabetes 51: 2929-2935. doi:10.2337/diabetes.51.10.2929. PubMed: 12351429. 12351429 10.2337/diabetes.51.10.2929

[49] Rosen ED, MacDougald OA (2006) Adipocyte differentiation from the inside out. Nat Rev Mol Cell Biol 7: 885-896. doi:10.1038/nrm2066. PubMed: 17139329. 17139329 10.1038/nrm2066

[50] Coenen KR, Gruen ML, Chait A, Hasty AH (2007) Diet-induced increases in adiposity, but not plasma lipids, promote macrophage infiltration into white adipose tissue. Diabetes 56: 564-573. doi:10.2337/db06-1375. PubMed: 17327423. 17327423 10.2337/db06-1375

[51] Lumeng CN, Bodzin JL, Saltiel AR (2007) Obesity induces a phenotypic switch in adipose tissue macrophage polarization. J Clin Invest 117: 175-184. doi:10.1172/JCI29881. PubMed: 17200717. 17200717 10.1172/JCI29881 PMC1716210

[52] Weisberg SP, McCann D, Desai M, Rosenbaum M, Leibel RL et al. (2003) Obesity is associated with macrophage accumulation in adipose tissue. J Clin Invest 112: 1796-1808. doi:10.1172/JCI19246. PubMed: 14679176. 14679176 10.1172/JCI19246 PMC296995

[53] Lunney JK (2007) Advances in swine biomedical model genomics. Int J Biol Sciences 3: 179-184. PubMed: 17384736. 10.7150/ijbs.3.179 PMC1802015 17384736

