# Supplementary figures and images for: MiRNA-181a Regulates Adipogenesis by Targeting Tumor Necrosis Factor-α (TNF-α) in the Porcine Model
Source: PLoS One. 2013 Oct 1;8(10):e71568. doi: 10.1371/journal.pone.0071568 (PMC3787936; doi:10.1371/journal.pone.0071568)

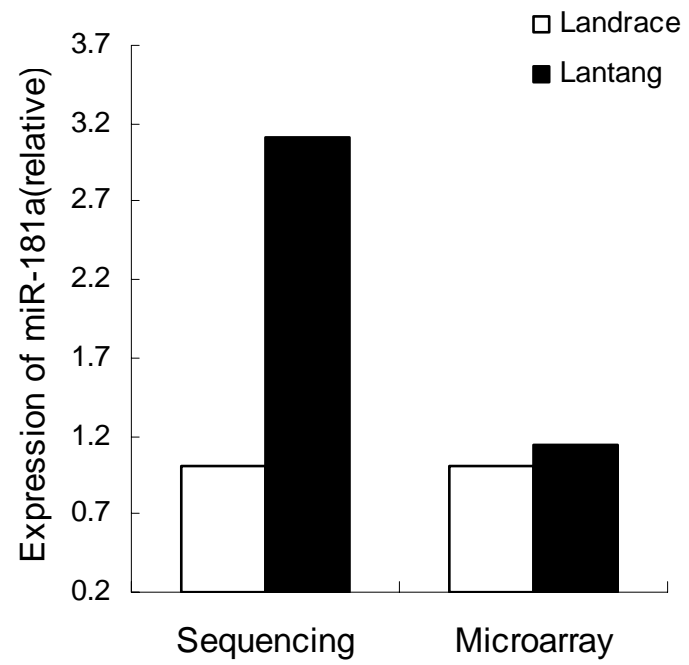

Supplement: Figure S1 — Expression of miR-181a in different breed of pigs. Total RNA were extracted from adipose tissues of Landrace and Lantang pigs (Fat-rich pigs) and subjected to miRNA sequencing and microarray. (PDF) [file pone.0071568.s001.pdf]
