# Supplementary material for: MiRNA-181a Regulates Adipogenesis by Targeting Tumor Necrosis Factor-α (TNF-α) in the Porcine Model
Source: PLoS One. 2013 Oct 1;8(10):e71568. doi: 10.1371/journal.pone.0071568 (PMC3787936; doi:10.1371/journal.pone.0071568)
Supplement: Table S1 — The primers of fat metabolism related genes. (DOC) [file pone.0071568.s002.doc]

Table S1 The primers of fat metabolism related genes

| **Accession No.** | **Gene** | **Primer sequence （5'to3'）** | | **Products（bp）** | **Tm（℃）** |
| --- | --- | --- | --- | --- | --- |
| EU012358 | GLUT1 | Forwards | GCGGTTTTCTATTACTCCACAAGC | 122 | 62 |
|  |  | Reverse | TCCACCACGAACAGCGACAC |  |  |
| EU590115 | GLUT4 | Forwards | TGAGTTTCCAGTATGTTGCG | 107 | 58 |
|  |  | Reverse | GGTTTCAGGCACTTTTAGGA |  |  |
| EF589048 | FASN | Forwards | ACCGAGTGGCTGGGTATT | 115 | 58 |
|  |  | Reverse | CAAGAAGAGGTTGTTGTGGG |  |  |
| XM003122963 | PED3B | Forwards | TCCTTGCCACAGACCTTA | 169 | 55 |
|  |  | Reverse | CTGGAGCTTTGCTAGTTGAG |  |  |
| EF601160 | adiponectin | Forwards | CGAGAAGGGTGAGAAAGGA | 153 | 58 |
|  |  | Reverse | GCTGAACGGTAGACATAGGC |  |  |
| EF583921 | ATGL | Forwards | GCGAAAATGTCATCATAACC | 175 | 53 |
|  |  | Reverse | ATGGTGCTCTTGAGTTCGT |  |  |
| AJ000482 | HSL | Forwards | GCCCGAGACGAGATTAG | 143 | 53 |
|  |  | Reverse | ATGAAGGGATTCTTGACG |  |  |
| X62984.1 | LPL | Forwards | AGGACACTTGCCATCTCATT | 127 | 55 |
|  |  | Reverse | GGGACCCAACTTTCATACAT |  |  |
| NM_214379 | PPARγ | Forwards | CATTCGCATCTTTCAGGG | 135 | 57 |
|  |  | Reverse | TGGACGCCATACTTTAGGA |  |  |
| DQ845171 | β-actin | Forwards | CCACGAAACTACCTTCAACTC | 131 | 56 |
|  |  | Reverse | TGATCTCCTTCTGCATCCTGT |  |  |
